# The Use of HIV Prevention Strategies and Services Reported by Black Women with a Risk for and with HIV in the United States

**DOI:** 10.1007/s10461-024-04578-7

**Published:** 2024-12-30

**Authors:** Toria Reaves, Rashunda Lewis, Sharoda Dasgupta, Shacara Johnson Lyons, Yunfeng Tie, Priya Nair, Tamara Carree, Xiaohong Hu, Jerris L. Raiford, Ruthanne Marcus

**Affiliations:** 1 Division of HIV Prevention, Centers for Disease Control and Prevention, National Center for HIV, Viral Hepatitis, STD, and TB Prevention, 1600 Clifton Road, NE, Atlanta, GA 30329 USA; 2 DLH Corporation, Atlanta, GA USA

**Keywords:** HIV, Black women, Health disparities/inequities, PrEP, HIV prevention

## Abstract

Black women are disproportionately affected by HIV. We analyzed data from two Centers for Disease Control and Prevention’s HIV surveillance systems to better understand HIV prevention strategies used by Black women at risk for and with HIV to help inform efforts to end HIV. Among sexually active Black women, we analyzed 2019 National HIV Behavioral Surveillance data on women without HIV (n = 4,033) and 2018–2020 Medical Monitoring Project data on women with HIV (n = 967). We reported percentages of HIV prevention strategies and services used and assessed differences between groups using Rao-Scott chi-square tests. Among Black women without HIV, 39% were aware of pre-exposure prophylaxis (PrEP); of these, 7% discussed PrEP with a healthcare provider, and 1% used PrEP in the past 12 months. Approximately 16% used a condom with their last sex partner; 36% reported that their last sex partner did not have HIV. Among Black women with HIV, 58% had condom-protected sex, 56% reported having sex while having sustained viral suppression, 3% had condomless sex with a partner on PrEP, and 24% had sex with a partner with HIV; 12% engaged in sex without using any HIV prevention strategy. HIV prevention strategies and services differed by selected demographic characteristics and social determinants of health. Although many sexually active Black women reported using HIV prevention strategies, there is room for improvement among those at risk for or with HIV. Tailoring prevention efforts based on individual needs and circumstances is essential for ending the HIV epidemic.

## Resumen

Las mujeres negras son afectadas de manera desproporcionada por el VIH. Analizamos datos de dos sistemas de vigilancia del VIH de los Centros para el Control y la Prevención de Enfermedades para comprender mejor las estrategias de prevención del VIH utilizadas por las mujeres negras en riesgo de contraer el VIH y con el VIH para ayudar a informar los esfuerzos para acabar con el VIH. Entre las mujeres negras sexualmente activas, analizamos los datos de la Vigilancia Nacional del Comportamiento del VIH de 2019 sobre mujeres sin VIH (n = 4,033) y los datos del Proyecto de Monitoreo Médico de 2018–2020 sobre mujeres con VIH (n = 967). Se informaron los porcentajes de las estrategias y los servicios de prevención del VIH utilizados, y se evaluaron las diferencias entre los grupos mediante las pruebas de Chi-cuadrado de Rao-Scott. Entre las mujeres negras sin VIH, el 39% conocía la profilaxis preexposición (PrEP); de estas, el 7% discutió la PrEP con un proveedor de atención médica, y el 1% usó la PrEP en los últimos 12 meses. Aproximadamente el 16% usó un condón con su última pareja sexual; El 36% reportó que su última pareja sexual no había tenido VIH. Entre las mujeres negras con VIH, el 58% tuvo relaciones sexuales protegidas con condón, el 56% reportó haber tenido relaciones sexuales mientras tenía supresión viral sostenida, el 3% tuvo relaciones sexuales sin condón con una pareja que tomaba PrEP y el 24% tuvo relaciones sexuales con una pareja con el VIH; El 12% mantuvo relaciones sexuales sin utilizar ninguna estrategia de prevención del VIH. Las estrategias y los servicios de prevención del VIH difieren según las características demográficas seleccionadas y los determinantes sociales de la salud. Aunque muchas mujeres negras sexualmente activas informaron haber utilizado estrategias de prevención del VIH, hay margen de mejora entre las que están en riesgo de contraer el VIH o con él. Para poner fin a la epidemia del VIH es esencial adaptar los esfuerzos de prevención en función de las necesidades y circunstancias individuales.

## Introduction

Progress has been made in reducing HIV transmission among women in the United States. The rate of HIV diagnoses among women aged ≥ 13 years declined by 12% overall and by 19% among Black/African American (hereafter referred to as Black) women aged ≥ 13 years from 2017 to 2021 [1]. Despite this decline, disparities persist, and compared with women of other races/ethnicities, Black women continue to be disproportionately affected by HIV. Of the 35,769 HIV diagnoses among persons aged ≥ 13 years in 2021, 41% (14,555) were among Black persons, and 24% (3544) of those were among Black women. Black women aged ≥ 13 years comprise 13% of the female population, but HIV infections among Black women accounted for 54% of 6584 diagnoses among women in 2021 [1, 2]. Heterosexual contact accounted for 90% of HIV diagnoses among Black women [1].

Because Black women are disproportionately affected by HIV, they are included as a priority population in the National HIV/AIDS Strategy (NHAS), the nation’s roadmap for ending HIV and ensuring that people with HIV live long and healthy lives [3]. Elevated HIV risk among Black women could be explained by multiple, interrelated individual and structural factors, including gender and race-based discrimination and stigma [4–7], limited or inequitable access to care [8], intimate partner violence and power imbalances in sexual relationships [4, 9–12], traumatic life experiences [13], male partners of unknown HIV status or risk behaviors [14, 15], exchanging sex for money or drugs [16], and increased rates of incarceration, homelessness, and poverty [4, 16–18]. Yet, despite the efficacy of biomedical preventive interventions, such as pre-exposure prophylaxis (PrEP), in reducing the risk of HIV acquisition and strategies to maintain viral suppression to prevent transmission and improve the quality of life for Black women with HIV, PrEP use among Black women who may benefit from it remains low [19–23]. Programs to address retention in care for Black women disproportionately affected by HIV and who are diagnosed with HIV that are tailored to transform and address the multiple challenges and historical trauma Black women face due to racism, sexism, classism, lack of social capital, compounded by HIV stigma and discrimination, are lacking [19–23]. Few evidence-based interventions have been developed to specifically address the HIV care and prevention needs of Black women with high incidence of and with HIV [1, 17, 24, 25].

Reducing HIV-related disparities—including those related to HIV transmission risk—requires employing a whole-person approach to the provision of HIV prevention, care, and treatment services [26]. A whole-person approach encourages health care providers to deliver a range of services (HIV testing, prevention, viral suppression, and other social services) appropriate to a person’s specific needs, regardless of HIV status, in order to promote optimal health and well-being. National data on the use of HIV prevention services—both for people from high incidence populations and with HIV—would help inform how HIV prevention efforts among Black women are used and can be improved. However, to our knowledge, no recent national estimates on the use of HIV prevention strategies and services among Black women are available. Recognizing the utility of using a whole-person approach in improving outcomes among Black women, we used two of the Centers for Diseases Control and Prevention (CDC’s) HIV surveillance systems—including the National HIV Behavioral Surveillance (NHBS) system and the Medical Monitoring Project (MMP)—to assess HIV prevention strategies and the use of prevention and care services among Black women with high incidence of, or with HIV, both overall and by demographic characteristics and social determinants of health (SDOH).

## Methods

NHBS is a national HIV surveillance system that focuses on describing behavioral risk factors for HIV, HIV testing behaviors, receipt of prevention services, and use of prevention strategies among populations with a high burden of HIV [27]. MMP is a national HIV surveillance system that describes representative estimates of demographic characteristics and SDOH, barriers to care and viral suppression, and clinical outcomes among people with HIV [28, 29]. NHBS and MMP are both cross-sectional surveys conducted as a part of routine public health surveillance and are considered non-research. Participating jurisdictions obtained local institutional review board approval to collect data when needed. Informed consent was obtained from all participants. For all analyses, we restricted our sample to women who reported their race as Black/African American, regardless of the report of any other race or ethnicity. Information about measures related to HIV prevention strategies is provided below. Additional details on SDOH included in this analysis can be found in Appendix Table 5 and 6.

### NHBS: Black Women at Increased Risk for HIV

#### Data Collection

We analyzed data from the 2019 cycle of NHBS among heterosexually active adults at increased risk for HIV (NHBS-HET) [27, 30]. Additional details regarding NHBS sampling methods and eligibility criteria have been described elsewhere [27]. In summary, NHBS-HET surveillance activities were conducted in 23 cities with high HIV prevalence. Participants in the 2019 NHBS-HET cycle were recruited using respondent-driven sampling (RDS) [31] and invited to participate in an anonymous interview and offered HIV testing. Interviewers administered the standardized survey, which collects information on demographics, substance use, HIV testing, general healthcare, and participation in HIV prevention activities. Participation in the 2019 NHBS-HET cycle was limited to persons who (1) were aged 18 to 60 years, (2) reported vaginal or anal sex with an opposite-sex partner in the 12 months before the interview, and (3) reported their gender as female or male.

#### Measures

We estimated the prevalence of behaviors associated with elevated HIV transmission risk and prevention strategies. Use of condoms during vaginal/anal sex was defined as responding ‘Yes’ to the use of condoms with the last male sexual partner in the past 12 months. Sex with a concordant HIV-negative partner was based on the report of the last male sex partner’s HIV status; respondents that selected ‘unknown’ were considered discordant. Prevention strategies included awareness of PrEP (yes/no). For those Black women who reported being aware of PrEP, we also ascertained if they had discussed PrEP with a healthcare provider in the past 12 months or had used PrEP in the past 12 months. Prevention strategies included receipt of free condoms from anyone other than a friend, relative, or sex partner (yes/no), participation in one-on-one conversations with an outreach worker, counselor, or prevention program worker about HIV prevention (yes/no), and participation in an organized session involving a small group of people to prevent HIV (yes/no) in the past 12 months.

#### Data Analysis

For this analysis, we included Black women who tested negative for HIV using an HIV test administered by project staff and who never injected any drugs other than those prescribed for them in order to focus on women who may acquire HIV via heterosexual contact and not drug use, and who were classified as having a low income. Low income was defined as having an income that was at or below 150% of the federal poverty level multiplied by a geographical adjustment, which was calculated as the ratio of the U.S. Census supplemental poverty measure threshold for the NHBS metropolitan statistical area (MSA) to the U.S. Census official poverty measure threshold. NHBS uses low income as a proxy for increased risk of acquiring HIV through heterosexual sex [27, 30].

For the 4033 sexually active, HIV-negative Black women meeting analysis criteria, we calculated the percentages reporting the use of HIV prevention strategies and services overall and by selected demographic characteristics and SDOH. We used Rao-Scott chi-square tests or Fisher’s exact tests (for small cell size) to assess differences between groups (*p* < 0.05).

### MMP: Black Women with HIV

#### Data Collection

Data from the 2018–2020 MMP cycles were collected annually from June of each cycle year through May of the following year. MMP uses a complex sample survey methodology with a 2-stage sampling design [29]. All persons with diagnosed HIV aged ≥ 18 years were eligible for participation. In the first stage, data reported for 16 states and Puerto Rico were sampled from all 50 U.S. states, the District of Columbia, and Puerto Rico. In the second stage, simple random samples of adults with diagnosed HIV were drawn from each participating jurisdiction from the National HIV Surveillance System (NHSS), a census of all persons with diagnosed HIV in the United States [32, 33]. The response rate was 100% at the first stage and ranged from 40–45% by cycle year at the second stage [28, 29].

Following informed consent, MMP staff interviewed respondents on demographic characteristics, SDOH, and HIV prevention strategies. Medical records were abstracted at respondents’ usual source of HIV care to collect data on clinical outcomes, including viral suppression status.

#### Measures

Sexually active Black women with HIV were asked about the following HIV prevention strategies: any condom-protected sex, sex while having sustained viral suppression, condomless sex with a partner with an HIV-negative or HIV-unknown status on PrEP, and sex with a partner with HIV. We also assessed whether women engaged in sex without the use of any HIV prevention strategy, defined as having vaginal or anal sex with at least one partner (1) who had an HIV-negative or unknown status, and the respondent did not have sustained viral suppression and (2) without a condom with at least one partner and the partner not reported to be taking PrEP. Sustained viral suppression was defined as having all viral loads undetectable or < 200 copies/mL in the past 12 months. PrEP use was assessed for the last five sexual partners.

Respondents were asked about receipt of HIV prevention services, including one-on-one HIV/STD risk-reduction conversations with a physician, nurse, or other health care worker; one-on-one HIV/STD risk-reduction conversations with an outreach worker, counselor, or prevention program worker; or receipt of free condoms from someone other than a friend, family member, or sex partner. All characteristics—including the use of HIV prevention strategies and services and SDOH—were based on the past 12 months unless otherwise noted.

#### Data Analysis

We analyzed data on 967 sexually active (defined as having anal or vaginal sex during the past 12 months) cisgender Black women with diagnosed HIV. We reported weighted percentages and corresponding 95% confidence intervals for Black women who used individual HIV prevention strategies and services overall and by selected demographic characteristics and SDOH. All estimates were weighted to adjust for nonresponse, and data were post-stratified to known population totals by age, race/ethnicity, and sex at birth from NHSS. We used Rao-Scott chi-square tests to assess differences between groups (*p* < 0.05).

We conducted all analyses using SAS (including SAS survey procedures), version 9.4 (SAS Institute, Cary, NC).

## Results

### NHBS: Black Women at Increased Risk for HIV

#### Demographic Characteristics and SDOH

Overall, 86% of Black women identified as non-Hispanic Black or non-multiracial Black (Table 1). Almost half (48%) were 18 to 34 years of age, 75% had at least a high school diploma or GED, 84% had an income at or below the federal poverty level, 33% were unemployed, 24% experienced homelessness in the past 12 months, 8% had been incarcerated in the past 12 months, and 20% experienced physical or sexual violence in the past 12 months. Eighty-three percent of Black women had any type of insurance, and among all participants in the analysis sample, 7% had any private insurance, and 75% had any public insurance.

**Table 1:** Prevalence of reported prevention strategies used by sexually active Black women at increased risk for HIV, overall and by demographic characteristics and social determinants of health—National HIV Behavioral Surveillance system, United States, 2019

	Overall		Condom-protected sex with last opposite-sex partner^a^		Last opposite-sex partner had a known HIV-negative status^b^					
	n	col %	n	row %	chisq	Chisq pvalue	n	row %	chisq	Chisq pvalue
Total	4033	100	634	16.0			1470	36.4		
Hispanic/Latina or multiracial identity^h,i^					3.6	0.05			7.7	0.01
Yes	581	14.1	76	13.3			182	31.4		
No	3452	85.6	558	16.5			1288	37.4		
Age, in years					19.0	0.0008			12.7	0.01
18–24	802	19.9	163	20.6			260	32.5		
25–34	1114	27.6	168	15.3			430	38.6		
35–44	861	21.3	123	14.5			340	39.5		
45–54	799	19.8	104	13.2			285	35.8		
55–60	457	11.3	76	17.0			155	33.9		
Educational attainment					8.2	0.02			14.0	0.0009
Less than high school	1007	25.0	138	14.1			320	31.8		
High school/GED	1986	49.2	306	15.6			740	37.3		
Greater than high school	1039	25.8	190	18.6			410	39.5		
Household income at or below the Federal Poverty Level					2.6	.10			10.3	0.001
Yes	3376	83.7	517	15.6			1194	35.4		
No	657	16.3	117	18.1			276	42.0		
Unemployed					0.6	.43			0.1	0.80
Yes	1338	33.2	432	16.3			986	36.6		
No^c^	2695	66.8	202	15.3			484	36.2		
Experienced homelessness in past 12 months^d^					0.3	.59			7.6	0.01
Yes	980	24.3	148	15.4			321	32.8		
No	3053	75.7	486	16.2			1149	37.7		
Incarcerated in the past 12 months^d, h, i^					1.7	.19			1.1	0.30
Yes	322	8.0	59	18.6			109	33.9		
No	3710	92.0	575	15.8			1361	36.7		
Experienced physical or sexual violence in past 12 months^d,e^					5.1	.02			27.9	< .0001
Yes	814	20.2	107	13.4			232	28.5		
No	3219	79.8	527	16.7			1238	38.5		
Had health Insurance					0.7	.40			15.6	< .0001
Yes	3338	82.8	534	16.2			1264	37.9		
No	690	17.1	100	14.9			206	29.9		
Type of Insurance (among all persons)										
*Any Private Insurance*^f^					1.9	.17			0.1	0.72
Yes	288	7.1	54	18.9			108	37.5		
No	3745	92.9	580	15.8			1362	36.4		
*Any Public Insurance*^g^					0.2	.70			12.6	0.0004
Yes	3040	75.4	475	15.9			1155	38.0		
No	993	24.6	159	16.4			315	31.8		

^a^Defined as use of a condom during the last vaginal or anal sex with the last opposite sex partner

^b^Respondents able to report their last opposite-sex partners’ HIV status as known HIV-positive, known HIV-negative, or unknown

^c^ Not unemployed includes those who are unable to work, students, retired persons, homemakers, those employed full-time or part-time, and those with another type of employment

^d^Past 12 months refers to the 12 months before the interview

^e^Physical violence is defined as being slapped, punched, shoved, kicked, shaken, or otherwise physically hurt in the past 12 months before the interview. Sexual violence is defined as being forced or pressured to have vaginal, oral, or anal sex when she did not want to in the past 12 months before interview

^f^Private insurance is defined as a private health plan – through an employer or purchased directly or TRICARE/CHAMPUS. Respondents could report > 1 type of health care coverage or insurance; not mutually exclusive groups

^g^Public insurance is defined as Medicaid, Medicare, some other government plan, or Veterans Administration coverage. Respondents could report > 1 type of health care coverage or insurance; not mutually exclusive groups

^h^Numbers may not add to total due to missing data

^i^Whole percentages may not add up to 100 due to rounding

#### Prevention strategies

Among HIV-negative Black women, 16% used a condom during vaginal or anal sex at the last sex encounter and 36% reported their last sex partner did not have HIV; Black women who identified as Hispanic/Latina or multiracial were less likely to have used a condom during vaginal or anal sex at the last sex encounter (13% vs. 17%; χ^2^ = 3.6, p = 0.05) and to report their last sex partner did not have HIV (31% vs. 37%; χ^2^ = 7.7, p = 0.01) (Table 1).

HIV prevention behaviors varied by selected demographic characteristics and SDOH. Use of a condom during last sex varied by age (18–24 years: 21%, 25–34 years: 15%, 35–44 years: 15%, 45–54 years: 13%, 55–60 years: 17%; χ^2^ = 19.0, p = 0.0008) and educational attainment (less than high school: 14%, high school/GED: 16%, greater than high school: 19%; χ^2^ = 8.2, p = 0.02). (Table 1). Black women who experienced physical or sexual violence in the past 12 months were less likely to report condom use at the last sex than those who did not (13% vs 17%; χ^2^ = 5.1, p = 0.02). Sex with a partner who had a known HIV-negative status also varied by age (18–24 years: 33%, 25–34 years: 39%, 35–44 years: 40%, 45–54 years: 36%, 55–60 years: 34%; χ^2^ = 12.7. p = 0.01) and educational attainment (less than high school: 32%, high school/GED: 37%, greater than high school: 40%; χ^2^ = 14.0, p = 0.0009). Compared with their counterparts, Black women with a household income at or below the federal poverty level (35% vs. 42%; χ^2^ = 10.3, p = 0.001) who experienced homelessness in the past 12 months (33% vs. 38%; χ^2^ = 7.6, p = 0.01), who experienced physical or sexual violence in the past 12 months (29% vs. 39%; χ^2^ = 27.9, p < 0.0001), or who did not have health insurance (30% vs. 38%; χ^2^ = 15.6, p < 0.0001) were less likely to report their last sex partner as having a known HIV-negative status. In particular, those without any public insurance were less likely to report that their last sex partner was HIV-negative than those with public insurance (32% vs. 38%; χ^2^ = 12.6, p = 0.0004).

Overall, 39% of Black women reported being aware of PrEP; of those that were aware, 7% discussed PrEP with a healthcare provider, and 1% used PrEP in the past 12 months. Black women who identified as Hispanic/Latina or multiracial were less likely to be aware of PrEP than monoracial Black women (28% vs. 40%; χ^2^ = 31.0, p < 0.0001) (Table 2). Some HIV prevention strategies varied by selected demographic characteristics and SDOH. PrEP awareness varied by age (18–24 years: 34%, 25–34 years: 41%, 35–44 years: 47%, 45–54 years: 37%, 55–60 years: 29%; χ^2^ = 52.5, p < 0.0001) and educational attainment (less than high school: 35%, high school/GED: 36%, greater than high school: 47%; χ^2^ = 43.0, p < 0.0001). PrEP awareness was lower among those who had a household income at or below the federal poverty level versus those who did not (37% vs. 46%; χ^2^ = 17.1, p < 0.0001), who did not experience homelessness in the past 12 months versus those who did (38% vs. 42%; χ^2^ = 5.6, p = 0.02), who did not experience physical or sexual violence during the past 12 months versus those who did (38% vs. 43%; χ^2^ = 7.2, p = 0.01), and did not have health insurance versus those who did (35% vs. 40%; χ^2^ = 5.7, p = 0.02). In particular, those without any public insurance were less likely to have PrEP awareness than those with public insurance (35% vs. 40%; χ^2^ = 7.1, p = 0.01).

**Table 2:** Prevalence of reported use of HIV prevention services among sexually active Black women without HIV, overall and by demographic characteristics and social determinants of health—National HIV Behavioral Surveillance system, United States, 2019

	Aware of PrEP				Aware of and discussed PrEP with a health care provider in past 12 months^f^				Aware of and used PrEP in past 12 months^f^			
	n	row %	chisq	Chisq pvalue	n	row %	chisq	Chisq pvalue	n	row %	chisq	Chisq pvalue
Total	1557	38.6			105	6.7			21	1.3		
Hispanic/Latina or multiracial identity			31.0	< .0001			2.7	0.10			0.2^i^	0.48
Yes	164	28.2			16	9.8			3	1.8		
No	1393	40.4			89	6.4			18	1.3		
Age, in years			52.5	< .0001			2.5	0.64			**	**
18–24	273	34.0			20	7.3			4	1.5		
25–34	456	40.9			32	7.0			9	2.0		
35–44	402	46.7			31	7.7			6	1.5		
45–54	294	36.8			15	5.1			1	0.3		
55–60	132	28.9			7	5.3			1	0.8		
Educational Attainment			43.0	< .0001			8.3	0.02			14.2	0.0008
Less than high school	356	35.4			36	10.1			12	3.4		
High school/GED	711	35.8			42	5.9			5	0.7		
Greater than High school	490	47.2			27	5.5			4	0.8		
Household income at or below Federal Poverty Level			17.1	< .0001			1.9	0.17			0.1^i^	0.40
Yes	1256	37.2			90	7.2			19	1.5		
No	301	45.8			15	5.0			2	0.7		
Unemployment			0.3	0.58			0.7	0.42			0.3	0.59
Yes	508	38.0			38	7.5			8	1.6		
No^a^	1049	38.9			67	6.4			13	1.2		
Experienced homelessness in past 12 months^b^			5.6	0.02			6.8	0.01			14.0	0.0002
Yes	409	41.8			39	9.5			13	3.2		
No	1148	37.6			66	5.7			8	0.7		
Incarcerated in past 12 months^b, g, h^			0.1	0.71			5.6	0.02			0.2^i^	0.2
Yes	127	39.0			15	11.8			3	2.4		
No	1429	38.5			90	6.3			18	1.3		
Experienced physical or sexual violence in past 12 months^c^			7.2	0.01			10.8	0.001			< .0001^i^	< .0001
Yes	347	42.7			37	10.7			13	3.8		
No	1210	37.6			68	5.6			8	0.7		
Health Insurance			5.7	0.02			1.2	0.27			0.1^i^	0.12
Yes	1318	39.5			85	6.4			15	1.1		
No	239	34.6			20	8.4			6	2.5		
Type of Insurance (among all persons)												
*Any Private Insurance*^*d*^			0.0009	0.98			1.9	0.17			0.2^i^	0.39
Yes	111	38.5			4	3.6				0.0		
No	1446	38.6			101	7.0			21	1.5		
*Any Public Insurance*^*e*^			7.1	0.01			0.1	0.70			0.2^i^	0.44
Yes	1209	39.8			80	6.6			15	1.2		
No	348	35.0			25	7.2			6	1.7		

	Received free condoms in past 12 months				Participated in individual HIV prevention intervention in past 12 months				Participated in group HIV prevention intervention in past 12 months			
	n	row %	chisq	Chisq pvalue	n	row %	chisq	Chisq pvalue	n	row %	chisq	Chisq pvalue
Total	1408	34.9			478	11.9			230	5.7		
Hispanic/Latina or multiracial identity			1.8	0.18			0.03	0.86			2.3	0.13
Yes	217	37.4			70	12.1			41	7.1		
No	1191	35.0			408	11.8			189	5.5		
Age, in years			14.1	0.01			5.5	0.24			8.9	0.06
18–24	317	40.0			104	13.0			49	6.1		
25–34	353	31.7			118	10.6			59	5.3		
35–44	306	35.5			102	11.9			43	5.0		
45–54	266	33.2			89	11.1			40	5.0		
55–60	166	36.3			65	14.2			39	8.5		
Educational Attainment			1.5	0.47			0.2	0.91			2.5	0.29
Less than high school	359	35.7			117	11.6			65	6.5		
High school/GED	675	34.0			234	11.9			102	5.1		
Greater than High school	374	36.0			127	12.2			63	6.1		
Household income at or below Federal Poverty Level			0.4	0.51			2.1	0.15			7.1	0.01
Yes	1186	35.1			411	12.2			207	6.1		
No	222	33.8			67	10.2			23	3.5		
Unemployment			3.6	0.06			0.4	0.51			0.1	0.74
Yes	494	37.0			165	12.3			74	5.5		
No^a^	914	34.0			313	11.6			156	5.8		
Experienced homelessness in past 12 months^b^			9.4	0.002			11.6	0.0007			8.2	0.0041
Yes	382	39.0			146	14.9			74	7.6		
No	1026	33.6			332	10.9			156	5.1		
Incarcerated in past 12 months^b, g, h^			15.8	< .0001			7.1	0.01			24.2	< .0001
Yes	145	45.0			53	16.5			38	11.8		
No	1262	34.0			425	11.5			192	5.1		
Experienced physical or sexual violence in past 12 months^c^			18.2	< .0001			2.7	0.10			6.1	0.01
Yes	336	41.3			110	13.5			61	7.5		
No	1072	33.3			368	11.4			169	5.3		
Health Insurance			13.7	0.0002			4.8	0.03			1.8	0.18
Yes	1209	36.2			413	12.4			198	5.9		
No	199	28.8			65	9.4			32	4.6		
Type of Insurance (among all persons)												
*Any Private Insurance*^*d*^			4.5	0.03			2.4	0.12			3.8	0.05
Yes	84	29.2			26	9.0			9	3.1		
No	1324	35.4			452	12.1			221	5.9		
*Any Public Insurance*^*e*^			20.9	< .0001			5.0	0.03			2.8	0.09
Yes	1121	36.9			380	12.5			184	6.1		
No	287	28.9			98	10.0			46	4.6		

^a^Not unemployed includes those who are unable to work, students, retired persons, homemakers, those employed full-time or part-time, and those with another type of employment

^b^Past 12 months refers to the 12 months before the interview

^c^Physical violence is defined as being slapped, punched, shoved, kicked, shaken, or otherwise physically hurt in the past 12 months before the interview. Sexual violence is defined as being forced or pressured to have vaginal, oral, or anal sex when she did not want to in the past 12 months before the interview

^d^Private insurance is defined as a private health plan—through an employer or purchased directly or TRICARE/CHAMPUS. Respondents could report > 1 type of health care coverage or insurance; not mutually exclusive groups

^e^Public insurance is defined as Medicaid, Medicare, some other government plan, or Veterans Administration coverage. Respondents could report > 1 type of health care coverage or insurance; not mutually exclusive groups

^f^Discussion with health care provider and use of PrEP asked only of participants who were aware of PrEP

^g^Numbers may not add to the total due to missing data

^h^Whole percentages may not add up to 100 due to rounding

^i^Fisher’s Exact Test used: cell counts too small to use Chi-square

^**^Cell size too small to calculate valid p-value

Among those who reported PrEP awareness, the prevalence of Black women who had PrEP discussions with a healthcare provider differed by educational attainment (less than high school: 10%, high school/GED: 6%, greater than high school: 6%; χ^2^ = 8.3, p = 0.02). PrEP discussions with a health care provider were less commonly reported among those who did not experience homelessness versus those who did (6% vs. 10%; χ^2^ = 6.8, p = 0.01), those who were not incarcerated versus those who were (6% vs. 12%; χ^2^ = 5.6, p = 0.02), and those who did not experience physical or sexual violence versus those who did (6% vs. 11%; χ^2^ = 10.8, p = 0.001). Among Black women aware of PrEP, less than 4% with any sociodemographic characteristic or SDOH reported PrEP use in the last 12 months.

#### Prevention Services

Overall, 35% of Black women reported receiving free condoms; 12% reported participating in one-on-one conversations with an outreach worker, counselor, or prevention program worker about HIV prevention in the past 12 months; and 6% reported participating in group HIV prevention intervention in the past 12 months (Table 2).

Receipt of free condoms by Black women varied by age (18–24 years: 40%, 25–34 years: 32%, 35–44 years: 36%, 45–54 years: 33%, 55–60 years: 36%; χ^2^ = 14.1, p = 0.01). Compared to their counterparts, Black women who were not employed (37% vs. 34%; χ^2^ = 3.6, p = 0.06), who did not experience homelessness (34% vs. 39%; χ^2^ = 9.4, p = 0.002), had not been incarcerated (34% vs. 45%; χ^2^ = 15.8, p < 0.0001), did not experience physical or sexual violence (33% vs. 41%; χ^2^ = 18.2, p < 0.0001), and did not have any health insurance (29% vs. 36%; χ^2^ = 13.7, p = 0.0002) were less likely to have received free condoms in the past 12 months. Those who had private insurance versus those who did not (29% vs. 35%; χ^2^ = 4.5, p = 0.03) and did not have public insurance versus those who did (29% vs. 37%; χ^2^ = 20.9, p < 0.0001) were also less likely to have received free condoms in the past 12 months.

In terms of HIV prevention interventions, Black women who did not experience homelessness (11% vs. 15%; χ^2^ = 11.6, p = 0.0007), had not been incarcerated (12% vs. 17%; χ^2^ = 7.1, p = 0.01), did not have health insurance (9% vs. 12%; χ^2^ = 4.8, p = 0.03), and did not have any public insurance (10% vs. 13%; χ^2^ = 5.0, p = 0.03) were less likely to participate in individual HIV prevention interventions than their counterparts. Black women who had a household income above the federal poverty (4% vs. 6%; χ^2^ = 7.1, p = 0.01), did not experience homelessness (5% vs. 8%; χ^2^ = 8.2, p = 0.0041), had not been incarcerated (5% vs. 12%; χ^2^ = 24.2, p < 0.0001), and did not experience physical or sexual violence versus did (5% vs. 8%; χ^2^ = 6.1, p < 0.0001) were less likely to participate in group HIV prevention interventions in the past 12 months.

### MMP: Black Women with HIV

#### Demographic Characteristics and SDOH

Overall, 12% of Black women identified as either Hispanic/Latino or multiracial, and over half (53%) were aged ≥ 45 years (Table 3). In addition, 21% had less than a high school education, 21% experienced unstable housing or homelessness during the past 12 months, 6% experienced physical violence by an intimate partner or forced sex during the past 12 months, 12% were uninsured during the past 12 months, and 44% received Ryan White HIV/AIDS Program (RWHAP) assistance during the past 12 months.

**Table 3:** Use of HIV prevention strategies reported by sexually active Black women with diagnosed HIV, overall and by demographic characteristics and social determinants of health—Medical Monitoring Project, United States, 2018–2020

	Sexually active Black women with HIV		Condom-protected sex^a^				Sex while having sustained viral suppression^b^				Condomless sex with a partner on PrEP^c^			
	n	wted col % (95% CI)	n	wted row % (95% CI)	chisq	Chisq pvalue	n	wted row % (95% CI)	chisq	Chisq pvalue	n	wted row % (95% CI)	chisq	Chisq pvalue
Overall	967		522	57.8 (54.5–61.2)			589	55.5 (50.7–60.3)			25	2.8 (1.8–3.9)		
Hispanic Latina/ multiracial identity					0.84	0.359			1.83	0.176			–	–
Yes	130	12.2 (7.8–16.7)	72	53.8 (44.6–63.1)			90	62.6 (51.1–74.1)			3	–		
No	837	87.8 (83.3–92.2)	450	58.4 (54.8–61.9)			499	54.6 (49.7–59.4)			22	2.7 (1.8–3.7)		
Age, in years					–	–			–	–			–	–
18–24	27	3.5 (2.0–5.0)	–	–			–	–			–	–		
25–34	143	16.0 (13.1–18.9)	66	45.8 (36.5–55.1)			69	42.7 (33.2–52.2)			8	–		
35–44	235	27.3 (23.6–30.9)	130	60.4 (53.7–67.1)			141	53.6 (46.2–61.1)			10	–		
45–54	319	32.3 (29.2–35.4)	175	58.2 (52.1–64.3)			203	57.8 (49.9–65.8)			3	–		
55–64	208	18.5 (15.8–21.2)	117	61.6 (52.9–70.2)			142	69.0 (61.3–76.6)			2	–		
≥65	35	2.5 (1.6–3.4)	20	60.4 (41.0–79.7)#			26	71.3 (53.8–88.8)#			0			
Educational attainment					2.72	0.257			3.61	0.164			–	–
Less than high school	219	21.2 (18.1–24.3)	128	63.5 (55.6–71.5)			125	51.3 (43.3–59.2)			2	–		
High School or equivalent	326	34.1 (30.7–37.6)	177	55.8 (50.6–61.0)			195	53.2 (46.3–60.1)			9	–		
Greater than high school	422	44.7 (40.4–49.0)	217	56.7 (51.2–62.1)			269	59.3 (52.8–65.9)			14	–		
Unstable housing or homelessness, past 12 months^e^					0.41	0.521			17.72	< .001			–	–
Yes	208	21.4 (18.4–24.3)	114	55.7 (48.2–63.2)			101	42.3 (34.8–49.8)			7	–		
No	759	78.6 (75.7–81.6)	408	58.4 (54.7–62.1)			488	59.1 (54.0–64.3)			18	2.3 (1.3–3.4)		
Unemployment^f^					0.01	0.908			1.98	0.159			–	–
Yes	136	14.1 (11.3–16.8)	69	58.3 (48.4–68.3)			74	48.0 (35.7–60.3)			5	–		
No	830	85.9 (83.2–88.7)	453	57.7 (54.4–61.0)			514	56.7 (51.9–61.5)			20	2.7 (1.5–3.8)		
Physical violence by an intimate partner or forced sex, past 12 months^g^					1.34#	0.248#			0.52#	0.471#			–	–
Yes	52	5.8 (3.7–7.8)	23	47.9 (29.8–66.1)#			29	49.4 (30.8–67.9)#			1	–		
No	896	94.2 (92.2–96.3)	490	58.4 (55.1–61.7)			546	55.6 (51.1–60.1)			22	2.5 (1.6–3.5)		
Health insurance or coverage for care or medications*					3.28	0.070			11.78	0.001			–	–
Yes	869	87.7 (83.9–91.5)	464	56.4 (53.1–59.7)			542	58.1 (53.6–62.6)			23	2.8 (1.8–3.8)		
No	93	12.3 (8.5–16.1)	56	68.5 (56.7–80.3)			44	39.5 (28.4–50.7)			1	–		
Type of health insurance or coverage for care or medications^h^														
Any Private					4.80	0.028			8.06	0.005			–	–
Yes	284	31.8 (27.7–35.9)	134	51.9 (46.1–57.6)			186	63.2 (56.1–70.3)			7	–		
No	662	68.2 (64.1–72.3)	376	60.3 (56.0–64.5)			389	52.2 (47.2–57.3)			17	2.8 (1.8–3.9)		
Any Public excluding Ryan White HIV/AIDS Program assistance only					0.15	0.702			0.09	0.766			–	–
Yes	657	65.3 (60.6–70.0)	361	58.4 (54.3–62.5)			400	55.4 (50.3–60.4)			17	2.9 (1.7–4.0)		
No	303	34.7 (30.0–39.4)	159	57.0 (51.1–62.9)			186	56.9 (48.0–65.7)			7	–		
Receipt of RWHAP assistance					0.78	0.377			5.19	0.023			–	–
Yes	437	43.9 (40.0–47.9)	235	55.5 (50.3–60.7)			281	62.2 (55.2–69.2)			12	–		
No	508	56.1 (52.1–60.0)	274	58.8 (54.1–63.5)			294	50.6 (43.9–57.2)			13	2.5 (1.5–3.6)		

	Sex with a partner with HIV^d^				Engaged in any sex without using an HIV prevention strategy, among sexually active persons			
	n	wted row % (95% CI)	chisq	Chisq pvalue	n	wted row % (95% CI)	chisq	Chisq pvalue
Overall	234	23.8 (20.9–26.6)			119	12.4 (9.8–15.1)		
Hispanic Latina/multiracial identity			1.21	0.271			–	–
Yes	33	28.4 (19.0–37.8)			11	–		
No	201	23.1 (20.1–26.1)			108	13.0 (10.3–15.7)		
Age, in years			–	–			–	–
18–24	–	–			–	–		
25–34	28	22.5 (14.6–30.5)			34	25.0 (16.7–33.3)		
35–44	49	19.8 (15.1–24.6)			29	10.8 (7.0–14.6)		
45–54	76	24.2 (19.2–29.2)			28	9.2 (5.8–12.7)		
55–64	64	30.4 (22.4–38.3)			20	8.8 (4.9–12.6)		
≥65	10	26.7 (11.6–41.7)#			2	–		
Educational attainment			2.52	0.284			13.18	0.001
Less than high school	47	23.3 (16.2–30.5)			31	14.6 (9.1–20.1)		
High School or equivalent	77	20.8 (16.1–25.5)			47	16.7 (12.1–21.3)		
Greater than high school	110	26.2 (22.2–30.3)			41	8.3 (5.8–10.9)		
Unstable housing or homelessness, past 12 months^e^			0.05	0.830			16.35	< .001
Yes	54	24.5 (16.8–32.2)			40	20.8 (14.2–27.4)		
No	180	23.6 (20.3–26.8)			79	10.2 (7.8–12.5)		
Unemployment^f^			1.98	0.159			3.84	0.050
Yes	29	18.7 (12.0–25.5)			24	17.3 (10.7–23.9)		
No	205	24.6 (21.4–27.9)			95	11.7 (9.1–14.2)		
Physical violence by an intimate partner or forced sex, past 12 months^g^			0.06	0.814			7.04	0.008
Yes	12	22.5 (11.8–33.2)			13	23.6 (11.1–36.0)		
No	219	23.9 (20.8–27.0)			105	11.9 (9.5–14.3)		
Health insurance or coverage for care or medications*			0.56	0.456			1.16	0.280
Yes	209	24.3 (21.1–27.4)			104	12.1 (9.0–15.1)		
No	24	20.9 (13.3–28.6)			15	16.3 (9.3–23.3)		
Type of health insurance or coverage for care or medications^h^								
Any Private			0.05	0.832			8.78	0.003
Yes	68	23.6 (19.0–28.1)			30	8.7 (5.5–11.9)		
No	163	24.2 (20.6–27.8)			88	14.4 (11.4–17.5)		
Any Public excluding Ryan White HIV/AIDS Program assistance only			0.06	0.812			1.18	0.278
Yes	157	23.5 (19.9–27.2)			85	13.7 (10.0–17.4)		
No	75	24.3 (19.1–29.5)			33	10.5 (6.5–14.4)		
Receipt of RWHAP assistance			0.97	0.325			2.09	0.148
Yes	119	25.7 (21.7–29.8)			47	10.9 (8.1–13.7)		
No	113	22.8 (18.8–26.9)			69	13.7 (10.1–17.3)		

*RWHAP* Ryan White HIV/AIDS Program

^a^Condoms were used with at least 1 vaginal or anal sex partner

^b^Defined as having all HIV viral loads being < 200 copies/mL or unsuppressed as documented in the medical record in the past 12 months before the interview

^c^At least 1 condomless-sex partner without HIV was on PrEP. PrEP use was only measured among the 5 most recent partners and was reported by the partner with HIV

^d^Sex with at least 1 partner with HIV

^e^Defined as experiencing unstable housing (i.e., moving in with others due to financial issues, moving 2 or more times, or being evicted) or homelessness (living on the street, in a shelter, in a single-room–occupancy hotel, or in a car) at any time during the past 12 months

^f^Unemployed persons included those who reported being unemployed at the time of the interview, excluding persons who were unable to work

^g^Physical violence by an intimate partner was defined as being slapped, punched, shoved, kicked, choked, or otherwise physically hurt by a romantic or sexual partner. Forced sex was based on reports of being threatened with harm or physically forced to have unwanted vaginal, anal, or oral sex

^h^Respondents could report > 1 type of health care coverage or insurance; not mutually exclusive groups

^*^Insured includes any type of insurance or coverage for care or medications, including private insurance, Medicaid, Medicare, or other public insurance. Those who received only RWHAP assistance, without coverage through any other insurance were considered not insured

Numbers may not add to total due to missing data

Whole percentages may not add up to 100 due to rounding

Note: Estimates with a coefficient of variation ≥ 0.30 and those based on a denominator sample size < 30 are considered unstable and are thus suppressed

^#^Estimates with an absolute CI width ≥ 30, estimates with an absolute CI width between 5 and 30 and a relative CI width > 130%, and estimates of 0% or 100% should be interpreted with caution. Any associated statistical testing should also be interpreted with caution

#### Prevention Strategies

Many HIV prevention behaviors were reported by sexually active Black women during the past 12 months, including 58% who had condom-protected sex, 56% who reported having sex while having sustained viral suppression, 3% who had condomless sex with a partner on PrEP, and 24% who had sex with a partner with HIV; 12% engaged in sex without using any HIV prevention strategy (Table 3). Some HIV prevention behaviors varied by selected SDOH. For instance, persons with any private insurance were less likely to have condom-protected sex than those who did not (52% vs. 60%; χ^2^ = 4.8, p = 0.028). Uninsured persons were less likely to have sex while having sustained viral suppression (40% vs. 58%; χ^2^ = 11.8, p = 0.001). People who experienced homelessness or other forms of unstable housing in the past 12 months (42% vs. 59%; χ^2^ = 17.7, p < 0.001), did not have any private insurance (52% vs. 63%; χ^2^ = 8.1, p = 0.005), or did not receive RWHAP assistance (51% vs. 62%; χ^2^ = 5.2, p = 0.023) were less likely to engage in sex while having sustained viral suppression than other persons. The prevalence of having sex without the use of any HIV prevention strategy was higher among those who experienced unstable housing or homelessness than those who did not (21% vs. 10%; χ^2^ = 16.3, p < 0.001), those who experienced physical violence by an intimate partner or forced sex than those who did not (24% vs. 12%; χ^2^ = 7.0, p = 0.008), and those who did not have any private insurance, compared with those who did (14% vs. 9%; χ^2^ = 8.8, p = 0.003).

#### Prevention Services

Overall, 67% reported having a one-on-one HIV/STD risk reduction conversation with a health care worker; 36% reported having a one-on-one HIV/STD risk reduction conversation with an outreach worker, counselor, or prevention program worker; and 49% received free condoms (Table 4).

**Table 4:** HIV prevention services received by sexually active Black women with diagnosed HIV, overall and by demographic characteristics and social determinants of health—Medical Monitoring Project, United States, 2018–2020

	One-on-one HIV/STD risk-reduction conversation with physician, nurse, or other health care worker				One-on-one HIV/STD risk-reduction conversation with outreach worker, counselor, or prevention program worker				Received free condoms			
	n	wted row % (95% CI)	chisq	Chisq pvalue	n	wted row % (95% CI)	chisq	Chisq pvalue	n	wted row % (95% CI)	chisq	Chisq pvalue
Overall	669	67.4 (63.0–71.8)			357	35.6 (31.9–39.3)			492	48.7 (43.9–53.5)		
Hispanic Latina/ multiracial identity			0.22	0.636			8.24	0.004			2.81	0.094
Yes	92	69.8 (58.6–81.0)			63	49.7 (37.8–61.6)			74	57.3 (46.2–68.3)		
No	577	67.1 (62.7–71.5)			294	33.6 (30.2–37.1)			418	47.5 (42.6–52.5)		
Age, in years			–	–			–	–			–	–
18–24	–	–			–	–			–	–		
25–34	102	68.7 (60.0–77.5)			59	40.0 (29.0–50.9)			63	41.6 (33.0–50.2)		
35–44	159	64.6 (55.7–73.6)			77	31.4 (25.6–37.1)			112	45.3 (36.3–54.4)		
45–54	219	67.1 (60.5–73.8)			111	34.0 (28.5–39.6)			184	55.5 (48.8–62.2)		
55–64	145	70.6 (62.9–78.2)			86	39.8 (31.9–47.8)			108	51.1 (43.0–59.2)		
≥65	22	62.9 (46.2–79.5)#			13	37.7 (21.0–54.3)#			13	41.1 (22.2–60.1)#		
Educational attainment			4.57	0.102			14.98	0.001			18.35	< .001
Less than high school	159	70.5 (63.6–77.4)			103	45.2 (38.1–52.3)			144	63.4 (55.9–71.0)		
High School or equivalent	237	70.7 (64.3–77.0)			132	39.1 (32.5–45.7)			165	49.4 (41.5–57.3)		
Greater than high school	273	63.6 (57.7–69.5)			122	28.4 (23.2–33.6)			183	41.3 (35.0–47.6)		
Homelessness, past 12 months^a^			0.56	0.455			3.02	0.082			2.47	0.116
Yes	65	72.3 (60.1–84.5)			42	45.6 (32.6–58.5)			53	58.5 (44.7–72.3)		
No	604	66.9 (62.1–71.7)			315	34.6 (30.9–38.2)			439	47.7 (43.0–52.4)		
Unstable housing or homelessness, past 12 months^b^			0.50	0.480			2.79	0.095			4.91	0.027
Yes	150	70.0 (63.1–76.8)			93	41.8 (32.5–51.0)			124	57.8 (49.0–66.6)		
No	519	66.8 (61.4–72.1)			264	33.9 (30.3–37.6)			368	46.3 (40.8–51.7)		
Unemployment^c^			0.17	0.682			0.04	0.843			0.12	0.733
Yes	97	69.2 (59.3–79.1)			49	36.4 (27.2–45.7)			66	50.2 (40.9–59.5)		
No	571	67.1 (62.6–71.6)			308	35.5 (31.7–39.3)			425	48.4 (43.2–53.7)		
Physical violence by an intimate partner or forced sex, past 12 months^d^			0.01#	0.944#			2.65#	0.103#			0.08#	0.778#
Yes	36	67.0 (49.1–84.9)#			28	48.6 (31.3–65.9)#			27	50.7 (33.9–67.6)#		
No	624	67.6 (63.2–72.1)			321	34.7 (30.9–38.4)			454	48.3 (43.2–53.3)		
Health insurance or coverage for care or medications*			0.10	0.755			2.35	0.126			2.81	0.094
Yes	598	67.3 (63.1–71.6)			314	34.5 (30.7–38.2)			433	47.7 (42.9–52.5)		
No	67	69.2 (57.3–81.2)			41	43.1 (32.2–53.9)			58	57.9 (46.0–69.7)		
Type of health insurance or coverage for care or medications^e^												
Any Private			0.27	0.601			4.65	0.031			17.36	< .001
Yes	188	66.6 (60.0–73.2)			84	29.4 (23.0–35.7)			107	38.2 (31.1–45.3)		
No	468	68.5 (63.7–73.2)			266	38.6 (33.9–43.2)			374	53.8 (48.7–58.9)		
Any Public excluding Ryan White HIV/AIDS Program assistance only			1.10	0.295			2.49	0.115			0.79	0.374
Yes	465	69.0 (64.4–73.6)			259	37.5 (33.1–41.9)			351	50.3 (45.2–55.5)		
No	198	64.7 (57.0–72.4)			94	31.6 (25.7–37.5)			139	46.4 (38.3–54.5)		
Receipt of RWHAP assistance			3.43	0.064			23.00	< .001			18.68	< .001
Yes	311	71.1 (66.2–76.1)			190	44.1 (39.0–49.1)			249	58.5 (52.0–65.0)		
No	345	65.6 (60.3–70.9)			160	29.5 (25.1–33.9)			231	41.3 (35.6–47.0)		

Numbers may not add to total due to missing data. Whole percentages may not add up to 100 due to rounding. Estimates with a coefficient of variation ≥ 0.30 and those based on a denominator sample size < 30 are considered unstable and are thus suppressed

^a^Defined as living on the street, in a shelter, in a single-room–occupancy hotel, or a car at any time during the past 12 months

^b^Defined as experiencing unstable housing (i.e., moving in with others due to financial issues, moving 2 or more times, or being evicted) or homelessness (living on the street, in a shelter, in a single-room–occupancy hotel, or in a car) at any time during the past 12 months

^c^Unemployed persons included those who reported being unemployed at the time of the interview, excluding persons who were unable to work

^d^Physical violence by an intimate partner was defined as being slapped, punched, shoved, kicked, choked, or otherwise physically hurt by a romantic or sexual partner. Forced sex was based on reports of being threatened with harm or physically forced to have unwanted vaginal, anal, or oral sex

^e^Respondents could report > 1 type of health care coverage or insurance; not mutually exclusive groups

^*^Insured includes any type of insurance or coverage for care or medications, including private insurance, Medicaid, Medicare, or other public insurance. Those who received only RWHAP assistance, without coverage through any other insurance were considered not insured

^#^Estimates with an absolute CI width ≥ 30, estimates with an absolute CI width between 5 and 30 and a relative CI width > 130%, and estimates of 0% or 100% should be interpreted with caution. Any associated statistical testing should also be interpreted with caution

The prevalence of having a risk reduction conversation with an outreach worker, counselor, or prevention program worker was lower among women who did not identify as Hispanic or being multiracial compared with those who did (34% vs. 50%; χ^2^ = 8.2, p = 0.004), those who did not have any private insurance compared with those who did (39% vs. 29%; χ^2^ = 4.7, p = 0.031), and those who did not receive RWHAP assistance compared with those who did (30% vs. 44%; χ^2^ = 23.0, p < 0.001). The prevalence of having a risk reduction conversation with an outreach worker, counselor, or prevention program worker also differed by educational attainment (less than high school: 45%, high school or equivalent: 39%; greater than high school: 28%; χ^2^ = 14.9, p = 0.001). There were no significant differences in one-on-one HIV/STD risk-reduction conversations by providers by selected demographic characteristics and SDOH.

The prevalence of receipt of condoms was lower among those who did not experience unstable housing or homelessness than those who did (46% vs. 58%; χ^2^ = 4.9, p = 0.027), those who did have any private insurance than those who did not (38% vs. 54%; χ^2^ = 17.4, p < 0.001), and those who did not receive RWHAP assistance than those who did (41% vs. 59%; χ^2^ = 18.7, p < 0.001). Receipt of free condoms varied by educational attainment (less than high school: 63%, high school or equivalent: 49%, greater than high school: 41%; χ^2^ = 18.4, p < 0.001).

## Discussion

To our knowledge, this is the first analysis that uses multiple national HIV surveillance systems to assess patterns in HIV prevention strategies and services used by Black women disproportionately affected by and with HIV in the United States. These findings demonstrate the need for tailored interventions focused on increasing access to HIV prevention services and strategies among Black women. Such efforts are essential to effectively reduce Black women’s risk factors for HIV, improve their overall health and well-being [3], and address the Ending the HIV Epidemic (EHE) goal of reducing new HIV infections in the United States by 90% by 2030 [34]. Our findings indicate that although some Black women are engaged in behaviors protective of their health and the health of their partners, many are not receiving HIV prevention services and strategies needed to decrease new HIV infections or prevent HIV transmission. Further, demographic characteristics and SDOH play an important role in how prevention services and strategies are accessed and utilized by Black women.

Other published literature has demonstrated gaps in the use of HIV prevention strategies and services, both among Black women affected by and with HIV. Essien et al. [35] conducted a qualitative study among economically disadvantaged Black women in Houston, Texas, and found that barriers to practicing safe sex included the belief that there was no risk based on their monogamous relationships, the lack of perception of the need to use condoms, lack of concern, being unprepared, and the partner’s refusal to use a condom. Kenya et al. [36] found that two-thirds of Black persons with HIV surveyed in Miami reported having sex without a condom despite knowing their HIV status. These findings demonstrate the need to focus on improving awareness of the risks of HIV transmission, access to services, and integrating HIV testing and care in a variety of settings, such as clinical settings, for all Black women.

Our findings extend the currently available research by examining how demographic characteristics and SDOH affect HIV prevention behaviors utilized by Black women and how this differs for Black women with and without HIV, demonstrating the need for a whole-person approach. For Black women without HIV, condom-protected sex and sex with a concordant partner were associated with both age and education. They were higher among those with higher incomes and who had not experienced violence. This finding is consistent with other studies that found intimate partner violence (IPV) and certain demographic characteristics, such as age and education, to be associated with an increased risk of HIV [10, 11, 37]. Those who had not experienced homelessness, had any health insurance, and had public health insurance were also more likely to report sex with a concordant partner. Structural factors such as these can either bolster or limit the ability of Black women in sexual relationships to feel empowered to discuss the use of prevention measures and to encourage their partners to be tested for HIV [16, 38].

Overall, more than one-third of Black women disproportionately affected by HIV reported being aware of PrEP. This finding is consistent with other studies that found similar rates of awareness among Black college women [39] and Black women at sexually transmitted infection (STI) clinics and emergency departments [20]. We found that PrEP awareness varied by demographic characteristics and SDOH.

Black women without HIV who experienced homelessness or IPV in the past 12 months were more likely to report PrEP awareness and use than Black women without HIV who did not experience these SDOH. These women may be linked to services that provide access to education and the provision of PrEP. This finding is supported by Bradley et al.[40] and Raiford et al. [16] that suggests that because people who are unstably housed may be living in high-risk environments associated with IPV and higher HIV transmission, they may need and use more HIV prevention strategies. Programs that address IPV as a contributor to sexual health risk may reduce HIV transmission [11].

In our study, PrEP use in the past 12 months was abysmally low at just over 1% of Black women among those who were aware of PrEP. Additionally, only 3% of Black women with HIV reported having condomless sex with a partner on PrEP. Other authors have found that the lack of PrEP acceptability and use may be attributed to racism, sexism, and economic inequities [41], concern about drug effects, access, cost, and adherence [21], and PrEP disapproval from others [42]. Studies suggest that women’s concerns, such as limited awareness, low perceived risk, side effects, costs, limited marketing, medical distrust, and PrEP education, are important drivers as Black women deliberate about PrEP as an HIV prevention strategy [20, 25, 43, 44]. Future interventions could include social networks, local organizations, and HIV prevention, treatment, and care outreach with specific strategies that are evidence-based address racism and cultural beliefs, and incorporate an approach that addresses social and structural determinants of health [25, 39–41]. Programs such as the #ShesWell: PrEP for Women (https://www.cdc.gov/stophivtogether/sheiswell/index.html #ShesWell: PrEP for Women | HIV Prevention | Let’s Stop HIV Together | CDC) initiative developed by the Black AIDS Institute (https://blackaids.org/campaign/black-women-and-prep/ Black Women and PrEP—Black AIDS Institute) that are designed by Black women for Black women and the HerPrEP (https://www.cdc.gov/hiv/funding/announcements/ps23-001/index.html PS23-001 | Announcements | Funding | HIV/AIDS | CDC) intervention will be essential for improving PrEP awareness and use among Black women. Healthcare providers could integrate routine behavioral assessments of sexual health into STI clinics, women’s health, and primary care settings—regardless of their race, gender, or personal circumstance—which could provide opportunities for patient education and referral to sexual health-related services and resources. Furthermore, the availability of long-acting injectable PrEP could be offered as an option for Black women interested in PrEP as an alternative to oral PrEP and can reduce barriers, such as stigma, to PrEP use and adherence [45].

For Black women with HIV, insurance status, particularly private health insurance, and RWHAP assistance were found to be associated with many prevention behaviors. Research shows that health insurance plays a vital role in patient care for all steps of the HIV continuum of care; people without health insurance are more likely to experience a lapse in HIV treatment and poor retention in care [46–48]. While this analysis did not examine the association of health insurance, quality of HIV care received, and patient-provider relationship with viral suppression, our findings suggest further exploration of these relationships in future studies is warranted.

Conversely, Black women with HIV were more likely to engage in sex without using an HIV prevention strategy if they had a high school education or less, if they experienced homelessness or unstable housing, were unemployed, had experienced physical or sexual violence in the past 12 months, or lacked private health insurance. These SDOHs can limit access to much-needed HIV prevention services. Black women with HIV may experience unmet needs for services and prevention education, which can increase transmission risk to their sexual partners. In addition to limited access to services, Black women with HIV may lack knowledge of HIV transmission, be less inclined to disclose their HIV status, and may delay linkage to care [49]. HIV-, racial-, and gender-based discrimination are also barriers to care [8]. Furthermore, Black women with HIV who have experienced IPV have been found to have less condom-protected sex, higher numbers of sexual partners, and poorer treatment adherence [10]; this may be due to an inability to negotiate prevention strategies used with abusive sexual partners [9, 10].

Participation in individual or group HIV prevention interventions is another strategy to reduce HIV transmission among Black women. In this analysis, we found that only 12% of Black women disproportionately affected by HIV had participated in individual HIV prevention interventions and only 6% had participated in group HIV prevention interventions. Settings such as homeless shelters or correctional facilities may provide education and services on HIV prevention, however, to reach more Black women, these services need to be widely available in the community, in family planning clinics, STI clinics, or in primary care settings where they are accessible to all Black women. One technique is to engage Black women with their peers using a group-centered approach, such as a "Sistah circle" proposed by Davis and colleagues [50].

CDC supports efforts to build the capacity of STI clinics to tailor strategies to provide PrEP and other HIV prevention services that are racially and gender concordant to improve access and education for Black women, specifically services that focus on Black women who have experienced IPV [10, 21]. Optimal HIV prevention strategies for Black women should focus on the importance of routine sexual health/SDOH screening in a culturally competent manner. These prevention strategies would increase opportunities for patient/provider engagement and education around sexual health and HIV/STI prevention, and patient intent (e.g., family planning, needs for sexual health services) and interventions that address concerns identified by Black women—poverty, gender inequality, stigma, and lack of women-controlled prevention technologies. When utilized, HIV prevention programs empower women to use their voices to advocate for condom use and HIV testing and take ownership of their health instead of fear and reliance on their partners to make decisions that impact their health and risk for HIV [4, 51].

### Limitations

This study had several limitations. First, the data from both surveillance systems are cross-sectional, and therefore, causality cannot be determined. Second, NHBS data were collected from urban areas with high HIV prevalence and may not be generalizable to all sexually active Black women in the US. Third, data on HIV prevention strategies and services are self-reported and may be subject to social desirability bias, resulting in over- or underestimates of these outcomes. Fourth, some prevalence estimates need to be interpreted with caution due to the small sample size for some subgroups. Further, while MMP response rates were suboptimal, MMP estimates were adjusted for nonresponse and post-stratified to population total by age, race/ethnicity, and sex from NHSS based on standard methodology.

## Conclusions

This analysis presents the use of HIV prevention strategies and services among Black women affected by HIV, overall and by selected demographic characteristics and SDOH. Although Black women use some HIV prevention strategies and services, there is room for improvement in meeting NHAS goals related to reducing HIV-related disparities and health inequities and to achieve EHE goals of reducing new HIV infections. Tailoring efforts to individual needs and addressing structural barriers are essential for reducing disparities related to HIV transmission risk, but also for improving the overall health and well-being of Black women affected by HIV.

## Appendix A

See Tables 5 and 6.

**Table 5:** Measures of demographic characteristics and social determinants of health – National HIV Behavioral Surveillance (NHBS)

Variable	Analytic categories	Interview question(s) corresponding to a variable	Additional notes
Gender Identity	Female	Do you consider yourself to be male, female or transgender?	Original categories for gender identity included: male, female, transgender Sex at birth not asked of respondents in 2019 NHBS interview
Age	18–24 years; 25–34 years; 35–44 years; 45–54 years; 55–60 years	N/A	Calculated based on date of birth and interview date NHBS-HET participants must be 60 years old or less to be eligible
Race/Ethnicity	Black, non-Hispanic, not multiracial; Black, Hispanic or multiracial	Which racial group or groups do you consider yourself to be in? You may choose more than one option Do you consider yourself to be of Hispanic, Latino/a, or Spanish origin?	Original categories for race included: American Indian or Alaska Native; Asian; Black or African American; Native Hawaiian or other Pacific Islander; White. Data on variables corresponding to race and Hispanic/Latino origin were used to identify those who had Hispanic or multiracial identity
Educational attainment	Less than high school; High school/GED; Greater than high school	What is the highest level of education you completed?	
Household income at or below the federal poverty level	Yes; No	What was your household income last year from all sources before taxes? Including yourself, how many people depended on this income?	2019 federal poverty level thresholds were calculated based on the HHS FPL guidelines: https://aspe.hhs.gov/topics/poverty-economic-mobility/poverty-guidelines/prior-hhs-poverty-guidelines-federal-register-references/2019-poverty-guidelines
Low income	N/A	N/A	Participants were classified as low income if their income was 150% of the federal poverty level multiplied by a geographical adjustment, which was calculated as the ratio of the U.S. Census supplemental poverty measure threshold for the NHBS city’s MSA^a^ to the U.S. Census official poverty measure thresholds^b^. The cities were adjusted thus: Multiplier—NHBS cities 1.0—Memphis; San Juan 1.1—Atlanta; Chicago; Dallas; Detroit; Houston; New Orleans; Virginia Beach; Portland 1.2—Baltimore; Denver; Miami; Philadelphia; Seattle 1.3—Boston; Nassau-Suffolk; New York City; Newark 1.4—Los Angeles; San Diego; Washington, DC 1.5—San Francisco
Unemployment	Unemployed; Not Unemployed (includes those who are unable to work, students, retired persons, homemakers, and those employed full time or part time)	What best describes your employment status?	
Homelessness	Yes; No	In the past 12 months, have you been homeless at any time? By homeless, I mean you were living on the street, in a shelter, in a Single Room Occupancy hotel (SRO), or in a car	
Incarceration	Yes; No	During the past 12 months, have you been held in a detention center, jail, or prison for more than 24 h?	
Physical or sexual violence	Yes; No	Physical violence: In the past 12 months, has anyone slapped, punched, shoved, kicked, shaken or otherwise physically hurt you? Sexual violence: In the past 12 months has anyone forced or pressured you to have vaginal, oral, or anal sex when you did not want to?	Physical or sexual violence was defined as reporting an experience of any of the following: physical violence alone, sexual violence alone, or both
Health Insurance	Yes; No	Do you currently have health insurance or health care coverage?	
Type of Insurance	Any Private Insurance; Any Public Insurance	What kind of health insurance or coverage do you currently have? - A private health plan—through an employer or purchased directly - Medicaid—for people with low incomes - Medicare—for the elderly and people with disabilities - Some other government plan - TRICARE/CHAMPUS - Veterans Administration coverage - Some other health insurance	People were considered to have any private insurance if they reported having a private health plan—through an employer or purchased directly or TRICARE/CHAMPUS People were considered to have any public insurance if they reported having Medicaid, Medicare, some other government plan, or Veterans Administration coverage

^a^U.S. Census Bureau. Table of supplemental poverty measure thresholds by metro area for 2017. https://www.census.gov/content/census/en/library/publications/2018/demo/p60-265.html. Revised September 2018. Accessed December 21, 2020

^b^U.S. Census Bureau. Table of poverty thresholds for 2017 by size of family and number of children. https://www.census.gov/data/tables/time-series/demo/income-poverty/historical-poverty-thresholds.html. Revised August 2020. Accessed December 21, 2020

**Table 6:** Measures of demographic characteristics and social determinants of health – Medical Monitoring Project (MMP)

Variable	Analytic categories	Interview question(s) corresponding to variable	Additional notes
Hispanic or multiracial identity	Yes, No	Which racial group or groups do you consider yourself to be in? You may choose more than one option Do you consider yourself to be of Hispanic, Latino/a, or Spanish origin?	Original categories for race included: American Indian or Alaska Native; Asian; Black or African American; Native Hawaiian or other Pacific Islander; White. Data on variables corresponding to race and Hispanic/Latino origin were used to identify those who had Hispanic or multiracial identity
Age	18–24 years, 25–34 years, 35–44 years, 45–54 years, 55–64 years, > = 65 years	N/A	Calculated based on date of birth and interview date
Educational attainment	Less than high school; high school or equivalent; more than high school	What is the highest level of education you completed?	
Homelessness, past 12 months	Yes, No	During the past 12 months, have you done any of the following: • Lived on the street? • Lived in a shelter? • Lived in a Single Room Occupancy (SRO) hotel? • Lived in a car?	People were considered to have experienced homelessness if they reported living on the street, shelter, SRO, or a car at any point during the past 12 months
Unstable housing or homelessness, past 12 months	Yes, No	During the past 12 months, have you done any of the following: • Lived on the street? • Lived in a shelter? • Lived in a Single Room Occupancy (SRO) hotel? • Lived in a car? During the past 12 months, did you ever move in with other people even for a little while because of financial problems? How many times have you moved during the past 12 months? Have you been evicted at any time during the past 12 months?	People were considered to have experienced unstable housing or homelessness if they reported: Experiencing unstable housing (i.e., moving in with others due to financial issues, moving 2 or more times, or being evicted), OR Homelessness (living on the street, in a shelter, in a single-room–occupancy hotel, or in a car) at any time during the past 12 months
Unemployment	Yes, No	Are you currently: • Employed for wages • Self-employed • Out of work • A homemaker • A student • Retired Unable to work	Unemployed persons included those who reported being unemployed at the time of the interview, excluding persons who were unable to work
Physical violence by an intimate partner or forced sex, past 12 months	Yes, No	During the past 12 months, has a partner slapped, punched, shoved, kicked, choked, or otherwise physically hurt you? During the past 12 months, have you been threatened or forced to have vaginal, anal, or oral sex when you did not want to?	People were considered to have experienced physical violence by an intimate partner or forced sex if they reported yes to either of the two questions
Any health insurance or coverage for care or medications	Yes, No	I’d like to ask you about all of the types of health insurance and coverage you had during the past 12 months. Please answer ‘yes’ or ‘no’ to each type. You may answer ‘yes’ to more than one type • Private insurance • Medicaid • Medicare • Coverage through the Ryan White HIV/AIDS Program or AIDS Drug Assistance Program, also known as ADAP • Tricare or CHAMPUS/CHAMPVA • Veterans Administration coverage • City, county, state, or other publicly funded insurance, not including Medicaid Any other insurance	People were considered to have any health insurance or coverage if they reported having private insurance, Medicaid, Medicare, or other public insurance. Those who received only RWHAP assistance without coverage through any other insurance were considered not insured
Type of health insurance or coverage for care or medications: Any private insurance	Yes, No	See above	
Type of health insurance or coverage for care or medications: Any public insurance (excluding Ryan White HIV/AIDS Program [RWHAP] only)	Yes, No	See above	People were considered to have any public insurance if they reported being covered through Medicaid, Medicare, or other public insurance—excluding those who received RWHAP assistance only
Receipt of RWHAP assistance	Yes, No	See above	People were considered to have received RWHAP assistance if they reported receiving coverage through the Ryan White HIV/AIDS Program or AIDS Drug Assistance Program, also known as ADAP
